# Osimertinib Rechallenge With Bevacizumab vs. Chemotherapy Plus Bevacizumab in EGFR-Mutant NSCLC Patients With Osimertinib Resistance

**DOI:** 10.3389/fphar.2021.746707

**Published:** 2022-01-03

**Authors:** Qingli Cui, Yanhui Hu, Qingan Cui, Daoyuan Wu, Yuefeng Mao, Dongyang Ma, Huaimin Liu

**Affiliations:** ^1^ Department of Integrated Traditional Chinese and Western Medicine, Affiliated Cancer Hospital of Zhengzhou University, Zhengzhou, China; ^2^ Nanjing University of Chinese Medicine, Nanjing, China; ^3^ Department of Medical Oncology, Affiliated Zhengzhou Central Hospital of Zhengzhou University, Zhengzhou, China; ^4^ Department of Pathology, Affiliated Cancer Hospital of Zhengzhou University, Zhengzhou, China; ^5^ Department of Medical Oncology, Second People’s Hospital of Pingdingshan, Pingdingshan, China

**Keywords:** osimertinib rechallenge, bevacizumab, EGFR, NSCLC, resistance

## Abstract

At present, treatment options for osimertinib resistance are very limited. Dual inhibition of the vascular endothelial growth factor (VEGF) and epidermal growth factor receptor (EGFR) significantly improved the progression-free survival (PFS) of advanced EGFR-mutant non–small cell lung cancer (NSCLC). After EGFR-tyrosine kinase inhibitor (TKI) resistance, EGFR-TKI continuation combined with VEGF inhibitors still had clinical benefits. It is unclear whether the addition of bevacizumab after osimertinib progresses will prolong the duration of the osimertinib benefit. We screened 1289 patients with NSCLC and finally included 96 patients to evaluate osimertinib combined with bevacizumab (osi + bev) versus chemotherapy combined with bevacizumab (che + bev) for patients with acquired resistance to osimertinib. The overall response rate (ORR) for osi + bev and chem + bev was 15.8% (6 of 38) and 20.7% (12 of 58), respectively. The median PFS for osi + bev and che + bev was 7.0 and 4.9 months (HR 0.415 95%CI: 0.252–0.687 *p* = 0.001). The median OS for osi + bev and che + bev was 12.6 and 7.1 months (HR 0.430 95%CI: 0.266–0.696 *p* = 0.001). Multivariate analyses showed that no brain metastases and osi + bev treatment after osimertinib resistance correlated with longer PFS (*p* = 0.044, *p* = 0.001), while the median PFS of osimertinib less than 6 months (*p =* 0.021) had a detrimental effect on sequent treatment. Only osi + bev treatment was identified as an independent predictor of OS (*p* = 0.001). The most common adverse events (AEs) of grade ≥3 were hypertension (13.2%) and diarrhea (10.5%) in the osi + bevacizumab group. Neutropenia (24.1%) and thrombocytopenia (19%) were the most common grade ≥3 AEs in the che + bev group. The overall incidence of serious AEs (grade ≥3) was significantly higher in the chemotherapy plus bevacizumab group. Our study has shown the superiority of osi + bev compared to che + bev after the failure of osimertinib, making it a preferred option for patients with acquired resistance to osimertinib.

## Introduction

Lung cancer is the second most commonly diagnosed cancer worldwide and the leading cause of cancer death (Sung et al., 2021). Non–small cell lung cancer (NSCLC) accounts for approximately 85% of all lung cancers (Planchard et al., 2018). Targeted drugs represented by epidermal growth factor receptor (EGFR)–tyrosine kinase inhibitors (TKIs) have brought revolutionary progress in the treatment of advanced NSCLC (Mitsudomi et al., 2010; Rosell et al., 2012; Mok et al., 2017; Soria et al., 2018). Unfortunately, no matter which generation of EGFR-TKI is used, patients will develop resistance sooner or later. Acquired EGFR exon 20 T790M mutation is the main mechanism of first-generation and second-generation EGFR-TKI resistance (Sequist et al., 2011). Osimertinib is an oral third-generation EGFR-TKI, which is effective against EGFR (exon 19 deletion or exon 21L858R) and exon 20 T790M mutation (Yver, 2016). Therefore, osimertinib can be used as the main treatment strategy after first- and second-generation EGFR-TKI resistance. Compared with the first- and second-generation EGFR-TKIs, first-line osimertinib application has longer progression-free survival (PFS) and overall survival (OS) (Soria et al., 2018; Ramalingam et al., 2020) and has a higher control rate of central nervous system metastases (Reungwetwattana et al., 2018). Therefore, it has been widely used in the first-line treatment of EGFR-sensitive mutations. With the extensive clinical application of osimertinib, acquired resistance has become a challenge faced by clinicians. Different from the first- and second-generation EGFR-TKIs, the resistance mechanism of osimertinib is complicated. Reported resistance mechanisms of osimertinib can be divided into EGFR pathway-dependent resistance (i.e., C797S or loss of T790M), upregulation of alternative signaling pathways (i.e., MET/HER-2 amplification and BRAF mutation), and histological transformation (Oxnard et al., 2018). C797S and MET amplification are the two most common resistance mechanisms, but effective drugs are not available in mainland China. For those who cannot access proper clinical trials, platinum-based chemotherapy with or without bevacizumab remains the standard treatment.

Several prospective randomized trials have established the benefit of the dual EGFR/VEGF pathway on PFS in advanced EGFR-mutant NSCLC (Nakagawa et al., 2019; Zhou et al., 2019; Makoto et al., 2020). VEGF levels were significantly increased in lung cancer cells and NSCLC tissues with EGFR mutations (Hung et al., 2016), and the enhanced expression of VEGF is frequently related to the resistance of EGFR-TKI (Hung et al., 2016). Anti-VEGF therapy combined with EGFR-TKI can reverse EGFR-TKI resistance (Byers and Heymach, 2007). However, it is unclear whether the addition of bevacizumab after the progress of osimertinib will re-sensitize the tumor to osimertinib. Therefore, this retrospective study compared osimertinib plus bevacizumab vs. chemotherapy plus bevacizumab in patients resistant to osimertinib.

## Materials and Methods

### Patients

We screened patients who received bevacizumab combined with osimertinib or chemotherapy after being resistant to osimertinib from April 2017 to January 2020 in the Affiliated Cancer Hospital of Zhengzhou University, Affiliated Zhengzhou Central Hospital of Zhengzhou University, and Second People’s Hospital of Pingdingshan. This retrospective study was approved by the ethics committee of the three participating institutions, without the need for informed consent. The inclusion criteria were as follows: 1) patients with stage IIIB or IV NSCLC with confirmed pathological diagnosis and classification; 2) EGFR-sensitive mutation or secondary T790M mutation; 3) ECOG score 0–2; 4) patients with at least a measurable lesion; and 5) normal bone marrow hematopoietic function and liver and kidney function. The exclusion criteria were as follows: 1) no T790M mutation after first-generation or second-generation EGFR-TKI resistance; 2) osimertinib combined with chemotherapy or radiotherapy or other targeted drugs; 3) patients with interstitial lung disease, radiation pneumonia, lung fibrosis, cardiac insufficiency, and history of deep vein thrombosis; 4) patients who did not respond at all to initial osimertinib; and 5) patients with incomplete efficacy evaluation or follow-up data.

### Treatment and Assessments

Eligible patients who received osimertinib combined with bevacizumab were defined as the osi + bev group, and those who received chemotherapy combined with bevacizumab were defined as the che + bev group. Patients in the osi + bev group received 80 mg of osimertinib daily combined with 7.5 mg/kg or 15 mg/kg of bevacizumab every 3 weeks. Patients in the che + bev group received chemotherapy combined with 7.5 mg/kg or 15 mg/kg of bevacizumab every 3 weeks. To assess the efficacy, a computed tomography scan of the chest and upper abdomen was evaluated every 6 or 9 weeks. If the patient had brain metastases, brain magnetic resonance imaging was evaluated too. Tumor response was evaluated according to the Response Evaluation Criteria in Solid Tumors (RECIST) (version 1.1) (Eisenhauer et al., 2009). PFS was calculated from the beginning of osi + bev or che + bev to disease progression or death. OS was defined as the time from osi + bev or che + bev to death or when the patients were censored at the last follow-up. Safety was monitored by medical records, blood tests, chief complaints, and physical examinations. Adverse events (AEs) were graded according to the National Cancer Institute Common Terminology Criteria for Adverse Events (NCI CTCAE) version 5.0.

### Statistical Analysis

Analyses were conducted using the GraphPad Prism version 5.0. Categorical variables are expressed as frequency (percentage) by descriptive methods. Chi-square tests (χ2 test) were used to compare clinical baseline characteristics between the two groups. PFS and OS were plotted using the Kaplan–Meier curve and compared using the log-rank test. *p* < 0.05 was considered statistically significant. Moreover, we used multivariate Cox regression models to estimate hazard ratio (HR) and exact 95% confidence intervals (CI) to analyze the prognostic factors.

## Results

### Patient Characteristics

**FIGURE 1:**
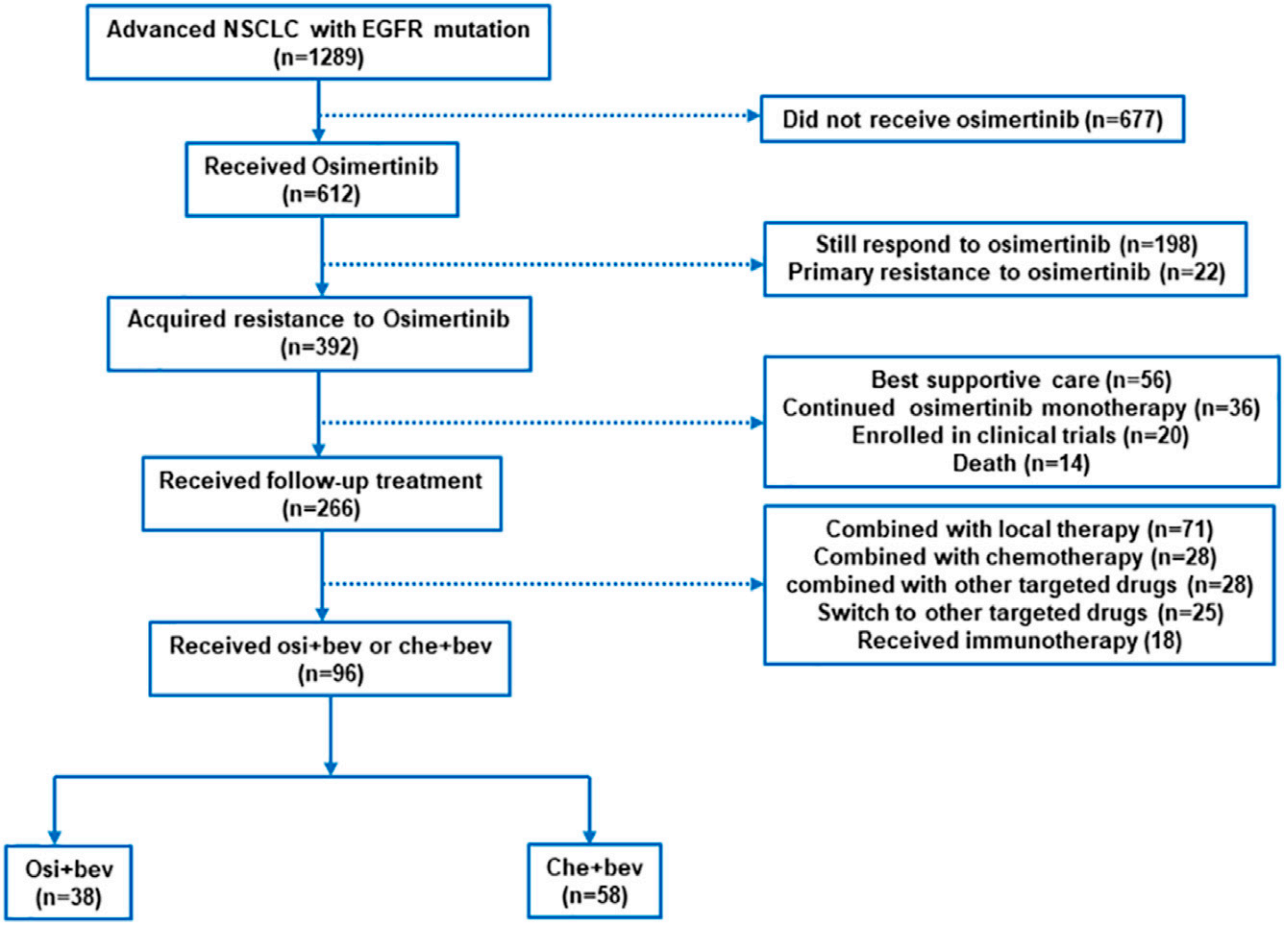
Study cohort selection.

Between April 2017 and January 2020, we screened 1289 patients from three institutions with advanced NSCLC, of which 96 were eligible for inclusion (Figure 1), specifically, the Affiliated Cancer Hospital of Zhengzhou University (*n* = 63), Affiliated Zhengzhou Central Hospital of Zhengzhou University (*n* = 17), and Second People’s Hospital of Pingdingshan (*n* = 16). All patients had lung adenocarcinoma and EGFR-sensitive mutation. The clinical characteristics of the patients are shown in Table 1. The median age was 57 years (range, 36–75). The majority of patients were women (74 of 96, 77.1%), had never smoked (78 of 96, 81.2%), and had a good ECOG performance status of 0 or 1 (79 of 96, 82.3%). Patients were previously treated with gefitinib (58 of 96, 60.4%) and icotinib (34 of 96, 35.4%) before osimertinib. In total, 4, 80, and 12 patients received osimertinib as first-, second-, and third-line treatment, respectively. All patients who received osimertinib in second- or third-line treatment had a T790M mutation. The median PFS for osimertinib was 9.5 months (95% CI: 7.1–12.2). In total, 76 (79.2%) patients had stage IV disease. Prior to the treatment of osimertinib, 20 (20.8%) patients had received chemotherapy and 4 (4.2%) had received bevacizumab. After the resistance of osimertinib, 38 cases received osimertinib (80 mg daily) rechallenge combined with bevacizumab (30 received 7.5 mg/kg and 8 received 15 mg/kg q3w), and 58 cases received chemotherapy combined with bevacizumab (46 received 7.5 mg/kg and 12 received 15 mg/kg). The platinum-doublet regimens that patients received in combination with bevacizumab were as follows: pemetrexed/carboplatin (29 of 58, 50%), paclitaxel/carboplatin (10 of 58, 17.2%), and gemcitabine/carboplatin (5 of 58, 8.6%). The single-agent chemotherapy regimens that patients received with bevacizumab were pemetrexed (9/58, 15.5%), docetaxel (3 of 58, 5.2%), and nab-paclitaxel (2 of 58, 3.4%). In total, 34 (35.4%) patients had brain metastases at diagnosis. At the time of osimertinib progression, an additional 19 patients developed brain metastases. Thus, before the start of bevacizumab plus chemotherapy or osimertinib, 55.2% of the patients had brain metastases.

**TABLE 1:** Baseline characteristics of patients.

Characteristics	Total (*n* = 96)	Osi + bev (*n* = 38)	Che + bev (*n* = 58)	*p*-value
Age				
Median (range)	57 (36–75)	56 (36–70)	57 (40–75)	0.139
≤ 65 years, n (%)	74 (77.1)	30 (78.9)	44 (75.9)	
> 65 years, n (%)	22 (22.9)	8 (21.1)	14 (24.1)	
Gender, n (%)				
Female	74 (77.1)	32 (84.2)	42 (72.4)	0.220
Male	22 (22.9)	6 (15.8)	16 (27.6)	
Smoking history, n (%)				
Never	78 (81.2)	33 (86.8)	45 (77.6)	0.296
Current/ever	18 (18.8)	5 (13.2)	13 (22.4)	
ECOG PS at progression, n (%)				
0–1	79 (82.3)	30 (78.9)	49 (84.5)	0.587
2	17 (17.7)	8 (21.1)	9 (15.5)	
EGFR mutation type at diagnosis, n (%)				
Exon 19 deletion	53 (55.2)	18 (47.4)	35 (60.3)	0.675
21 L858R	43 (44.8)	20 (52.6)	23 (39.7)	
Brain metastasis at diagnosis, n (%)				
Yes	34 (35.4)	14 (36.8)	20 (34.5)	0.527
None	62 (64.6)	24 (63.2)	38 (65.5)	
Brain metastasis after osimertinib, n (%)				
Yes	53 (55.2)	23 (60.5)	30 (51.7)	0.675
None	43 (44.8)	15 (39.5)	28 (48.3)	
Stage at diagnosis, n (%)				
IIIB	20 (20.8)	8 (21.1)	12 (20.7)	0.966
IV	76 (79.2)	30 (78.9)	46 (79.3)	
Number of treatment lines of osimertinib				
1	4 (4.2)	1 (2.6)	3 (5.2)	0.426
2	80 (83.3)	34 (89.5)	46 (79.3)	
3	12 (12.5)	3 (7.9)	9 (15.5)	
PFS of osimertinib, n (%)				
≤6 months	36 (37.5)	18 (47.4)	18 (31)	0.133
>6 months	60 (62.5)	20 (52.6)	40 (69)	
Prior chemotherapy, n (%)				
Yes	20 (20.8)	8 (21.1)	12 (20.7)	0.966
No	76 (79.2)	30 (78.9)	46 (79.3)	
Prior bevacizumab therapy, n (%)				
Yes	4 (4.2)	0	4 (6.9)	0.098
No	92 (95.8)	38 (100)	54 (93.1)	

EGFR, epidermal growth factor receptor; osi + bev, osimertinib plus bevacizumab; che + bev, chemotherapy plus bevacizumab; PFS, progression-free survival.

### Effectiveness

**FIGURE 2:**
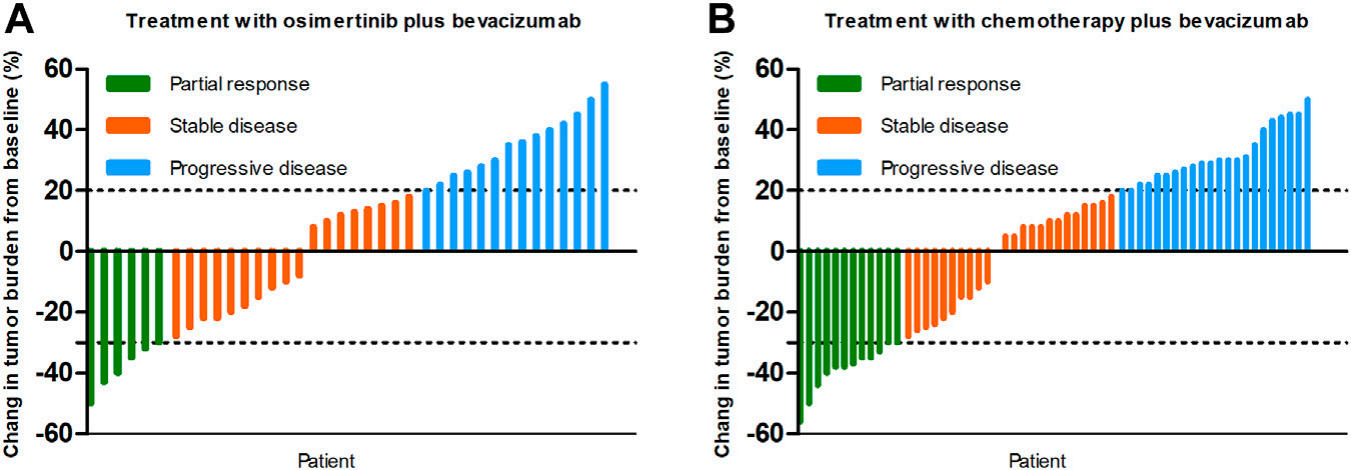
Best response in tumor burden from baseline in the two groups.

**FIGURE 3:**
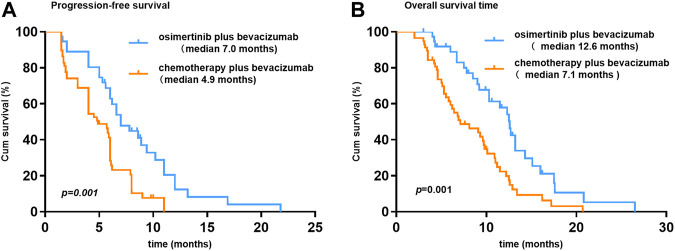
Kaplan–Meier curves of progression-free survival and overall survival.

**FIGURE 4:**
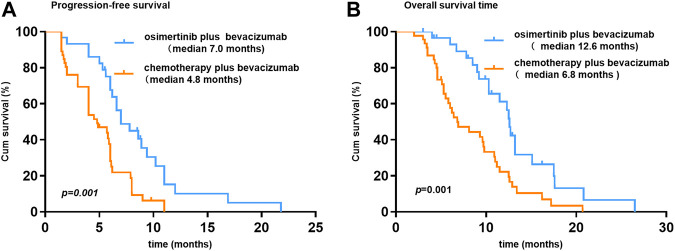
Kaplan–Meier curves of progression-free survival and overall survival for patients who received bevacizumab at 7.5 mg/kg.

At the time of data cutoff (March 1, 2021), the median follow-up time was 19.5 months (range, 6.9–35.9 months). No complete response cases were observed in the two groups. A total of 38 patients were evaluable for efficacy: 6 patients with partial response (PR), 18 with stable disease (SD), and 14 with progressive disease (PD) in the osi + bev group. On the other hand, there were 12 patients with PR, 24 with SD, and 22 with PD in the che + bev group. The overall response rate (ORR) for osi + bev and che + bev was 15.8% (6 of 38) and 20.7% (12 of 58), respectively (Figure 2). The median PFS for osi + bev and che + bev was 7.0 and 4.9 months, respectively (HR 0.415 95% CI: 0.252–0.687 *p* = 0.001) (Figure 3A). The median OS for osi + bev and che + bev was 12.6 and 7.1 months, respectively (HR 0.430 95% CI: 0.266–0.696 *p* = 0.001) (Figure 3B). When we excluded the 15 mg/kg bevacizumab dosage, we found that the PFS and OS of the two groups were very close to that of the total population. The median PFS for osi + bev and che + bev was 7.0 and 4.8 months, respectively (HR 0.398 95% CI: 0.228–0.694 *p* = 0.001) (Figure 4A). The median OS for osi + bev and che + bev was 12.6 and 6.8 months, respectively (HR 0.397 95% CI: 0.227–0.694 *p* = 0.001) (Figure 4B).

### Multivariate Analyses of PFS and OS

To further identify the risk factors related to PFS or OS, we used multivariate Cox regression analysis to determine protective or adverse prognostic factors. As for PFS, multivariate Cox regression analyses suggested that no brain metastases (HR 0.528, 95% CI: 0.283–0.984, *p* = 0.044) and osi + bev treatment (HR 0.403, 95% CI: 0.233–0.697, *p* = 0.001) were considered as protective prognosis factors, while the median PFS of osimertinib of less than 6 months (HR 1.861, 95% CI: 1.099–3.149, *p* = 0.021) had a detrimental effect on the subsequent treatment. As for OS analysis, patients without brain metastases showed a marginally longer OS than those with brain metastases, but it was not statistically significant. Only osi + bev treatment remained as an independent predictor of OS (*p =* 0.001) (Table 2).

**TABLE 2:** Multivariate analyses of progression-free survival and overall survival to assess the impact factor.

	PFS		OS	
	HR (95%CI)	*p*-value	HR (95%CI)	*p*-value
Age (≤ 60 vs. > 60)	1.287 (0.753–2.200)	0.356	1.187 (0.682–2.066)	0.545
Gender (female vs. male)	0.954 (0.467–1.652)	0.940	0.943 (0.502–1.771)	0.635
Smoking (no vs. yes)	0.963 (0.254–3.656)	0.956	0.657 (0.134–2.118)	0.542
ECOG (0 vs. 1–2)	0.713 (0.371–1.367)	0.308	0.702 (0.381–1.586)	0.322
EGFR mutation (21L858 vs. 19del)	0.670 (0.405–1.108)	0.118	0.684 (0.189–3.909)	0.146
Brain metastases (none vs. yes)	0.528 (0.283–0.984)	0.044	0.531 (0.302–1.160)	0.058
PFS of osimertinib (≤ 6 months vs. > 6 months)	1.861 (1.099–3.149)	0.021	1.258 (0.807–2.169)	0.379
Treatment after osimertinib resistance (osi + bev vs. che + bev)	0.403 (0.233–0.697)	0.001	0.395 (0.225–0.692)	0.001

EGFR, epidermal growth factor receptor; osi + bev, osimertinib plus bevacizumab; che + bev, chemotherapy plus bevacizumab; PFS, progression-free survival; OS, overall survival.

### AEs

Table 3 summarizes the major adverse events. Most AEs were generally mild (grade < 3). Proteinuria was the most common AE of the osi + bev group (34.2%). Neutropenia was the most frequent AE of the che + bev group (50%). Grade ≥3 AEs included hypertension (13.2%), diarrhea (10.5%), thrombocytopenia (7.9%), proteinuria (7.9%), anemia (7.9%), neutropenia (5.3%), liver function disorder (5.3%), anorexia (5.3%), and hypoproteinemia (2.6%) in the osi + bev group. Neutropenia (24.1%), thrombocytopenia (19%), fatigue (17.2%), anemia (12.1%), liver function disorder (10.3%), hypertension (10.3%), anorexia (10.3%), proteinuria (8.6%), hypoproteinemia (8.6%), and nausea (6.9%) were the most common AEs of grade ≥3 in the che + bev group. The overall incidence of serious AEs (grade ≥ 3) was significantly higher in the che + bev group.

**TABLE 3:** Summary of adverse events. Values are expressed as frequencies [n (%)].

Adverse event	Osi + bev (*n* = 38)		Che + bev (*n* = 58)	
	All grades	Grade ≥ 3	All grades	Grade ≥ 3
Proteinuria	13 (34.2)	3 (7.9)	17 (29.3)	5 (8.6)
Thrombocytopenia	12 (31.6)	3 (7.9)	15 (25.9)	11 (19)
Neutropenia	9 (23.7)	2 (5.3)	29 (50.0)	14 (24.1)
AST/ALT elevation	8 (21.1)	2 (5.3)	14 (24.1)	6 (10.3)
Hypoproteinemia	10 (26.3)	1 (2.6)	16 (27.6)	5 (8.6)
Anemia	7 (18.4)	3 (7.9)	13 (22.4)	7 (12.1)
Fatigue	8 (21.1)	0	19 (32.8)	10 (17.2)
Rash	7 (18.4)	0	2 (3.4)	0
Anorexia	8 (21.1)	2 (5.3)	15 (25.9)	6 (10.3)
Nausea	7 (18.4)	0	16 (27.6)	4 (6.9)
Vomiting	3 (7.9)	0	10 (17.2)	2 (3.4)
Headache	4 (10.5)	0	5 (8.6)	0
Oral mucositis	5 (13.2)	0	7 (12.1)	0
Pharyngodynia	3 (7.9)	0	6 (10.3)	0
Hypertension	11 (28.9)	5 (13.2)	18 (31)	6 (10.3)
Diarrhea	9 (23.7)	4 (10.5)	14 (24.1)	2 (3.4)
Bleeding	8 (21.1)	2 (5.3)	12 (20.7)	1 (1.7)

WBC, white blood cell; ALT, alanine aminotransferase; AST, aspartate aminotransferase; osi + bev, osimertinib plus bevacizumab; che + bev, chemotherapy plus bevacizumab.

## Discussion

While osimertinib has achieved outstanding efficacy in EGFR-mutant NSCLC in terms of PFS and OS, most people inevitably develop resistance, which presents another challenge in the treatment of NSCLC. The resistance mechanism of osimertinib is heterogeneous and mostly non-targeted, including a higher proportion of EGFR-independent mechanisms than EGFR-dependent mechanisms (Leonetti et al., 2019; Mehlman et al., 2019; Zhao et al., 2019). Although important research has been conducted on the mechanism and treatment strategies of osimertinib resistance, there is still no unified standard treatment method for osimertinib resistance, and the efficacy is not satisfactory. For erlotinib combined with osimertinib in the treatment of patients with EGFR C797S found alongside T790M, the PFS was only about three months (Wang et al., 2017). For c-Met inhibitor combined with osimertinib in the treatment of c-Met-driven acquired resistance, the longest PFS was close to 5 months (Sequist et al., 2020).

Aside from targeted therapy, chemotherapy and the addition of antiangiogenic inhibitors to EGFR-TKI therapy after progression are also alternative strategies. Studies have shown that patients with resistance to osimertinib derived prolonged clinical benefits from the continuous use of osimertinib alone or in combination with radiotherapy or chemotherapy (Le et al., 2018; Su et al., 2021; White et al., 2021). However, it has not been elucidated whether the combination of osimertinib and bevacizumab is beneficial after the progress of osimertinib. The rationale behind this study is based on the fact that VEGF expression levels change dynamically during anti-tumor therapy, and EGFR-TKI resistance is often accompanied by increased levels of VEGF (Takeuchi et al., 2012). VEGF signaling plays an important role in the formation of new blood vessels, and inhibition of VEGF is a key therapeutic strategy for cancer treatment (Manzo et al., 2017). EGFR mutation enhances VEGF expression in lung cancer (Hung et al., 2016). Preclinical and clinical data support dual inhibition of EGFR and VEGF in NSCLC with EGFR mutations as a promising strategy to improve patient prognosis (Le et al., 2021). Compared with treatment with EGFR-TKI alone, dual inhibition of VEGF and EGFR significantly improved the PFS of advanced EGFR-mutant NSCLC in first-line treatment (Landre et al., 2020; Peravali et al., 2020). Even after EGFR-TKI resistance, EGFR-TKI continuation combined with VEGF inhibitors still had clinical benefits (Otsuka et al., 2015; Jiang et al., 2017). Dual inhibition of VEGF and EGFR pathways had the potential advantage of reversing EGFR-TKI resistance (Larsen et al., 2011). A retrospective analysis of patients with advanced NSCLC who failed osimertinib treatment showed that osimertinib rechallenge combined with apatinib reached a median PFS of 4 months (95% CI: 3.5–4.5 months) (Yang et al., 2021). How about the combination of osimertinib and bevacizumab in osimertinib resistance?

This is the first study to compare the efficacy of adding bevacizumab to osimertinib versus chemotherapy plus bevacizumab after the progression of osimertinib. Although the ORR of che + bev was better than that of osi + bev (20.7 vs. 15.8%), the median PFS of che + bev was shorter than that of osi + bev (7 months in osi + bev vs. 4.9 months in che + bev, HR 0.415 95% CI: 0.252–0.687 *p* = 0.001). The benefits of ORR in che + bev have not been translated into prolongation of OS (12.6 months in osi + bev vs. 7.1 months in che + bev, HR 0.430 95% CI: 0.266–0.696 *p* = 0.001), as exhibited in Table 2. As for PFS, multivariate Cox regression suggested that no brain metastases (HR 0.528, 95% CI: 0.283–0.984, *p* = 0.044) and osi + bev treatment (HR 0.403, 95% CI: 0.233–0.697, *p* = 0.001) were considered as protective prognosis factors, while the median PFS of osimertinib of less than 6 months (HR 1.861, 95% CI: 1.099–3.149, *p* = 0.021) had a detrimental effect on subsequent treatment. As for OS, the median PFS of osimertinib of less than 6 months was an adverse prognosis factor, but there was no statistically significant difference. Only osi + bev treatment remained an independent predictor of OS (HR 0.395, 95% CI: 0.225–0.692, *p* = 0.001).

Bevacizumab and osimertinib act on different pathways, one regulating angiogenesis and one inhibiting tumor growth. Theoretically, receiving both drugs may confer additional benefits to NSCLC. So far, whether osimertinib combined with chemotherapy or bevacizumab has a synergistic effect is still controversial. In patients with advanced NSCLC with EGFR T790M, a randomized phase II trial showed a median PFS of 15.8 months for osimertinib monotherapy and 14.6 months for osimertinib and carboplatin-pemetrexed combination therapy, indicating no synergistic effect of combination (Tanaka et al., 2021). On the contrary, another study suggests that osimertinib combined with chemotherapy can be beneficial to patients after the progression of multi-line therapy (White et al., 2021). A single-arm study reported that among 49 NSCLC patients with EGFR mutation, osimertinib plus bevacizumab showed an ORR of 80% and a median PFS of 19 months (Yu et al., 2020). Another single-arm prospective trial of osimertinib plus bevacizumab in 14 patients with leptomeningeal metastasis (LM) from EGFR mutation showed an LM ORR of 50%, median PFS of 9.3 months, median OS of 12.6 months, and one-year survival rate of 35.7% (Lu et al., 2021). On the contrary, another phase II study showed that, compared to osimertinib monotherapy, although ORR was slightly better in the osimertinib and bevacizumab combination arm, combination therapy could not show advantages in PFS and OS, even in subgroup analyses (Akamatsu et al., 2021). The results of our study contradict this phase II study. The reason is unclear. We speculate that the timing of treatment, different populations, and drug doses may affect the research results. The dose of bevacizumab used in this phase II clinical study was 15 mg/kg, while most patients in our study received 7.5 mg/kg. Clinical trials did not find any significant difference between the two doses of bevacizumab on the PFS and OS of NSCLC. However, it still lacks a large-scale phase III study to confirm it. Considering economic factors or adverse reactions, it is very common for clinicians to choose the 7.5 mg/kg dose of bevacizumab. VEGF and EGF share a common downstream signaling pathway and may be independent of each other during tumorigenesis and acquired treatment resistance. The anti-VEGF inhibitor may induce a different tumor environment. Theoretically, the tumor environment is different in osimertinib-naive and pre-treated patients, and excessive inhibition of VEGF may affect the efficacy of EGFR-TKI. We are not sure whether the dose of bevacizumab affects the synergy with osimertinib. This needs further experimental verification. In addition, we need further research to confirm the optimal combined dose of osimertinib and bevacizumab, which will be synergistic and will not cause serious adverse reactions.

A study reported that in patients with EGFR mutations, compared with first-line chemotherapy without TKI, front-line EGFR-TKI significantly reduces the sensitivity of subsequent chemotherapy (Zeng et al., 2014). In our study, osi + bev showed more clinical benefits than che + bev in patients with osimertinib acquired resistance, which may be related to the reduced efficacy of chemotherapy after EGFR-TKIs treatment. On the other hand, regardless of frequency or severity, compared with che + bev, the adverse reactions of osi + bev are milder. The osi + bev combination is superior to che + bev in terms of safety because it avoids the toxicities of chemotherapy. Bevacizumab has its own series of adverse reactions, such as hypertension and proteinuria, which were observed in the two groups. The reduction in the dose of bevacizumab greatly reduced the incidence of adverse effects. In the osi + bev group, hypertension (13.2%), diarrhea (10.5%), proteinuria (7.9%), thrombocytopenia (7.9%), and anemia (7.9%) were the most AEs of grade 3 or higher. The adverse reactions related to bone marrow suppression were obviously higher in the che + bev group than in the osi + bev group. This supports the fact that the addition of bevacizumab to osimertinib would be more tolerable than chemotherapy and bevacizumab for patients resistant to osimertinib. Despite efforts to adjust multiple factors through Cox regression analysis and exclude patients with primary resistance to osimertinib, this retrospective analysis still has some limitations. First, retrospective research may inevitably introduce collection bias. In the case of significant progression and clinical deterioration, physicians had to choose to stop osimertinib. Patients who have progressed in imaging evaluation and have no clinical symptoms are more inclined to choose combination or switch to other treatments rather than continuing to use osimertinib alone. Second, we included patients who received different chemotherapy regimens and different bevacizumab dosages. The treatment of patients is not uniform, which may affect the results. Third, histomolecular profiles at progression should be collected to distinguish the benefits of people under different resistance mechanisms. Furthermore, other strategies in the following treatment, such as immunotherapy checkpoint inhibitors, the addition of local therapy, or radiotherapy, should be evaluated, which will inevitably affect OS analysis.

In conclusion, although this study is a retrospective study, the efficacy of osi + bev and che + bev was compared in patients with osimertinib resistance. Compared with che + bev, osi + bev provides significantly longer PFS and OS, and the toxicity is tolerable. This observation indicates that for patients who have failed osimertinib treatment, especially for those with a non-targetable resistance mechanism, bevacizumab plus continuous osimertinib should be considered an appropriate regimen. This program is worthy of large-scale verification in randomized clinical trials.

## Data Availability

The original contributions presented in the study are included in the article/Supplementary Material; further inquiries can be directed to the corresponding author.
